# Correlations Between MR Apparent Diffusion Coefficients and PET Standard Uptake Values in Simultaneous MR-PET Imaging of Prostate Cancer

**DOI:** 10.3390/ijms26030905

**Published:** 2025-01-22

**Authors:** Andrii Pozaruk, Vitaliy Atamaniuk, Kamlesh Pawar, Alexandra Carey, Jeremy Cheng, Marian Cholewa, Jeremy Grummet, Zhaolin Chen, Gary Egan

**Affiliations:** 1 Department of Photomedicine and Physical Chemistry, Institute of Medical Sciences, The Medical College of The University of Rzeszów, 35-310 Rzeszów, Poland; 2 Institute of Physics, College of Natural Sciences, University of Rzeszów, 35-959 Rzeszów, Poland; vitaliya@dokt.ur.edu.pl (V.A.); mcholewa@ur.edu.pl (M.C.); 3 Monash Biomedical Imaging, Monash University, Clayton, VIC 3168, Australia; kamlesh.pawar@monash.edu (K.P.); alexandra.carey@monash.edu (A.C.); zhaolin.chen@monash.edu (Z.C.); gary.egan@monash.edu (G.E.); 4 Monash Institute of Cognitive and Clinical Neurosciences and School of Psychological Sciences, Monash University, Clayton, VIC 3168, Australia; 5 Monash Imaging, Monash Health, Clayton, VIC 3168, Australia; 6 Department of Surgery, Central Clinical School, Monash University, Melbourne, VIC 3004, Australia; jeremycheng1996@gmail.com (J.C.); jpgrummet@gmail.com (J.G.); 7 Department of Data Science and AI, Faculty of Information Technology, Monash University, Clayton, VIC 3168, Australia

**Keywords:** standardized uptake values, apparent diffusion coefficient, prostate cancer, simultaneous PET/MR, deep learning, attenuation correction, prostate-specific membrane antigen

## Abstract

This study evaluated the hypothesis that ^68^Ga-PSMA-11 PET SUV, obtained via an advanced DL approach, correlates better with MR ADC maps than values from conventional PET-MR. Additionally, we aimed to identify the optimal SUV threshold for maximum correlation with ADC values. A cohort of 32 prostate cancer patients underwent CT and corresponding PET-MR imaging. The dataset underwent K-fold cross-validation, dividing it into four folds. In each fold, 24 patients were used for training, and 8 for validation to create DL models. ADC maps from 27 out of 32 patients were successfully aligned with T2 images for detailed analysis, revealing an inverse correlation (ρ = −0.20 to −0.51) between ADC and SUV values in prostate cancer zones. Statistically significant differences in mean SUV values were observed between PETMRI and PETDL. DL-based SUV values show a stronger correlation with ADC than conventional PET-MR values in our investigation.

## 1. Introduction

Prostate cancer is the fifth leading cause of cancer death in the world [1]. Imaging modalities such as PET-MRI, PET-CT, and multiparametric MRI are essential for cancer staging and diagnosis in prostate cancer [2]. These methods complement traditional PSA testing and biopsy for more precise detection and evaluation [3].

However, PSA testing has significant limitations, such as its association with overdiagnosis and overtreatment [4]. Elevated PSA values are not specific to prostate cancer and can result from benign conditions like prostatitis or benign prostatic hyperplasia [5]. These limitations underscore the importance of pre-biopsy imaging techniques, such as multiparametric MRI, which can improve diagnostic accuracy, reduce unnecessary biopsies, and better characterize prostate lesions [6].

For prostate cancer, metastases are most commonly found in the bone, lymph nodes (LN), liver, and thorax [7]. Magnetic resonance imaging has a limited value for detecting the LN and distant metastasis. For this reason, a new whole-body PET technique based on radioisotope ^68^Ga-PSMA tracer(s) can be used. It provides higher accuracy for the identification of metastasis in LN and the whole body, as well as for detecting cancer relapse [8,9]. In contrast to other methods, ^68^Ga-PSMA can more accurately detect prostate cancer [10].

Similarly, to the ^68^Ga-PSMA method, multiparametric MR images based on T2-weighted imaging, MR spectroscopic imaging, and DWI are powerful tools for diagnosing malignant tumors [11]. The DWI method measures random water movement, defined as ADC values, which are inversely correlated with cellularity and reflect tumor microstructure [12,13,14]. Prostate malignancy shows lower intensity in ADC images compared to benign lesions, and ADC values can be used as indicators of therapeutic response, tumor behavior, and disease-free intervals [15,16]. Additionally, ADC values are inversely correlated with Gleason scores, providing insights into cancer characteristics and aggressiveness [17,18,19].

New PET-MR systems have enabled combining PET and DWI imaging with high-resolution T2-weighted prostate imaging [20]. The PET-MR scanner allows for the quantitative assessment of prostate cancer at the molecular and metabolic levels in one examination and is highly accurate in detecting primary lesions [21]. Histogram analyses of images may also aid in cancer detection by enabling estimation of ADC parameters such as median ADC, kurtosis, skewness, and entropy [22]. Combinations of SUV and ADC values can increase the diagnostic accuracy of hybrid PET-MRI. While some studies report an inverse correlation between PET and ADC parameters in prostate cancer [23,24], others show weak or no correlation [25,26].

Several comparative analyses of PET and DWI have been presented for hybrid PET-MRI systems [27,28]. Wetter et al. [26] showed an inverse correlation between SUV and ADC values in bone metastases from prostate cancer using [18F] choline PET-MRI. Wang et al. [27] demonstrated that SUV_max_/ADC ratio values could combine MRI with ^68^Ga-PSMA-11 PET-CT for detecting prostate cancer. Meyer et al. [29] linked entropy from ADC maps to Total Lesion Glycolysis (TLG) and Metabolic Tumor Volume in head and neck squamous cell carcinomas. Bruckmann et al. [28] showed that diffusion restriction and SUV_max_ are associated with tumor grading, aiding in predicting neuroendocrine neoplasm grade in ^68^Ga-DOTATOC PET-MRI. However, PET data accuracy relies on precise AC μ-maps, which can vary SUV values by up to 5% in the body and 31% in bone lesions [30,31]. Accurate AC μ-maps are crucial for PET image reconstruction in prostate cancer management.

In recent years, deep learning (DL) has emerged as a transformative approach in medical imaging. DL refers to a subset of artificial intelligence that employs neural networks to analyze complex datasets, enabling advancements in imaging techniques and data processing. DL methods have shown promising results in estimating PET AC μ-maps based on paired MRI and CT data [32,33].

This study aimed to evaluate the correlation between DL-derived SUV and ADC values in prostate cancer zones and compare them to conventional imaging methods. Additionally, it sought to establish the optimal SUV threshold for maximum correlation with ADC values, providing insights into tumor aggressiveness and diagnostic utility.

## 2. Results

To support the aims of this research, detailed studies of CT, MR, and DL methods using mask thresholding, statistics, and correlation analyses between ADC and SUV values were performed. The PET-MRI technique proved to be highly sensitive in the identification of various prostate cancer lesions in PZ and TZ. A dataset of twenty-seven patients was used to analyze the correlation between ADC and SUV. The cancer was detected in transitional zones in ten patients, and in peripheral zones in seventeen patients.

**Figure 1:**
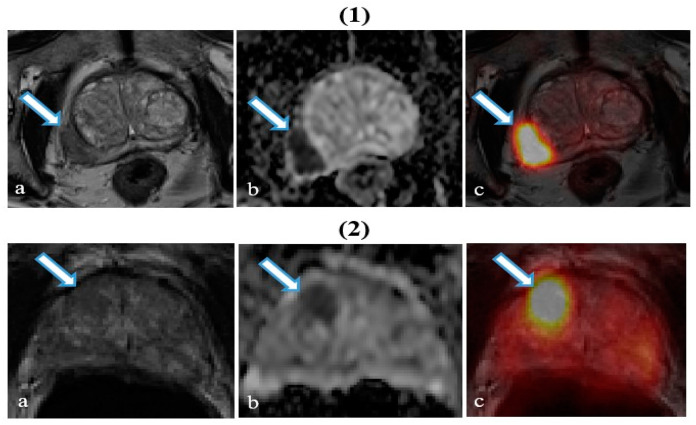
Patients with prostate cancer to the left of the peripheral zone with a Gleason score 4 + 5 = 9 (**1**) and transitional zone with a Gleason score 3 + 4 = 7 (**2**). The arrows in the axial section point to areas of cancer. (**a**) T2-weighted image showing a poorly defined cancerous lesion. (**b**) ADC map with a corresponding cancer lesion. (**c**) Overlapped T2-weighted and reconstructed PET images.

The PET parameters in PZ for the patient presented in Figure 1 were as follows: SUV_mean_ = 16.75, SUV_max_ = 25.32, SUV_min_ = 8.93, SUV_median_ = 16.23, SUV_kurtosis_ = −0.57, and SUV_skewness_ = 0.47. The corresponding ADC parameters (×10^−3^ mm^2^ s^−1^) were as follows: ADC_mean_ = 0.66, ADC_max_ = 1.56, ADC_min_ = 0.31, ADC_median_ = 0.59, ADC_kurtosis_ = 1.33, and ADC_skewness_ = 1.30. The PET parameters in the TZ were as follows: SUV_mean_ = 6.10, SUV_max_ = 9.14, SUV_min_ = 3.96, SUV_median_ = 5.83, SUV_kurtosis_ = −0.63, SUV_skewness_ = 0.59. The ADC values were as follows: ADC_mean_ = 0.95, ADC_max_ = 1.55, ADC_min_ = 0.64, ADC_median_ = 0.91, ADC_kurtosis_ = −0.04, and ADC_skewness_ = 0.67. In Figure 1, the axial T2-weighted MRI image (column a) shows a low signal intensity lesion compared to the ADC map (column b). The ADC map presents diffusion restrictions in peripheral and transitional zones, while in the PET image overlapped over the T2-weighted image (column c), a higher activity in peripheral and transitional zones is observed.

**Figure 2:**
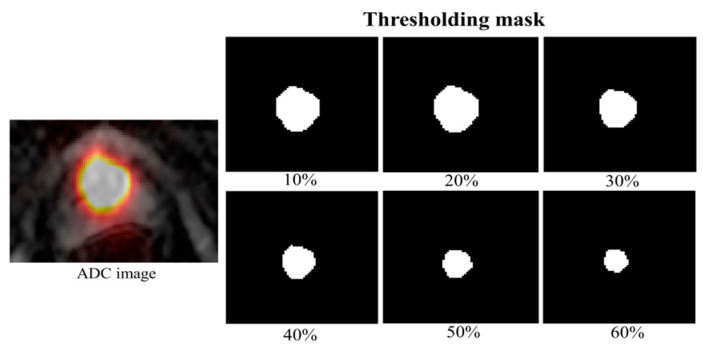
ADC map overlaid over the PET image of the prostate cancer colored in Hot (Left column). Six different ROI masks for cancer were created by applying a percentage threshold of SUV_max_ values of 10%, 20%, 30%, 40%, 50%, and 60% (Right column).

**Figure 3:**
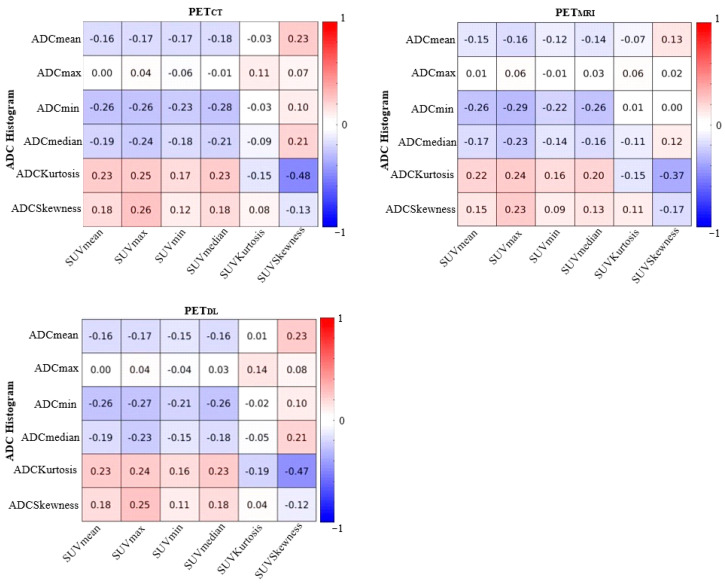
Correlations between ADC and SUV values for 27 test subjects with cancer in peripheral and transitional zones calculated using ROI mask of prostate cancer.

**Figure 4:**
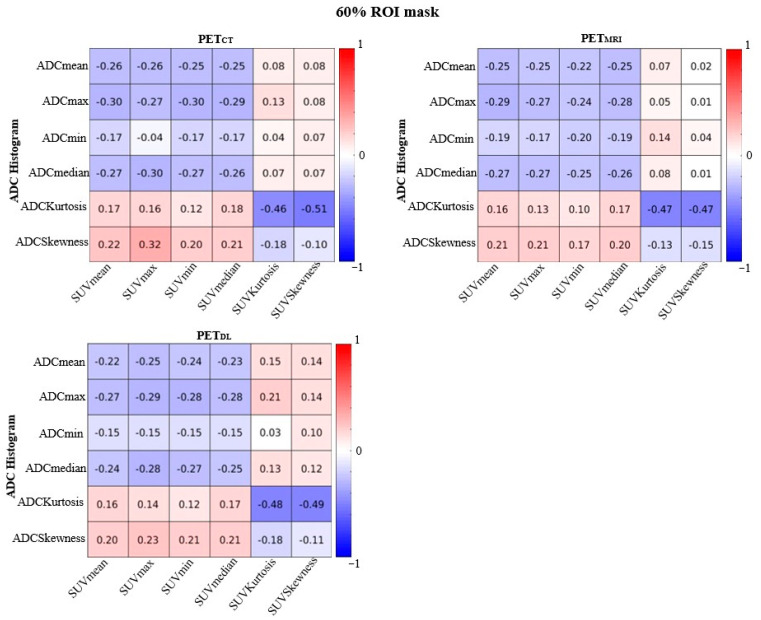
Correlations between ADC and SUV values for 27 test subjects with cancer in peripheral and transitional zones calculated using the ROI mask of prostate cancer with thresholding by 60% of the ROI mask for measuring SUV and ADC values.

Spearman’s correlation coefficients between PET and ADC parameters were calculated for cancer ROIs in the PZ and TZ using thresholded ROI masks. These masks were generated by applying percentage thresholds of SUVmax values ranging from 10% to 60%, as illustrated in Figure 2. The results are displayed as heat maps and shown in Figure 3 and Figure 4.

Figure 3 and Figure 4 show the correlation coefficients between ADC and PET images using an ROI mask of prostate cancer and an ROI mask of prostate cancer with thresholding by 60%, estimating the ADC and SUV values in the ROIs of cancer in TZ and PZ for 27 subjects. There were no significant correlations between PET and MRI parameters for cancer in PZ and TZ zones. The correlation coefficient between ADC and SUV values in Figure 3 was ρ = −0.48 for ADC_Kurtosis_ and SUV_Skewness_, presenting the expressed difference between PET_DL_ and PET_MRI_ values. The correlations between ADC_Kurtosis_ and SUV_Skewness_ for each method were as follows: ρ = −0.48 for PET_CT_, ρ = −0.37 for PET_MRI,_ and ρ = −0.47 for PET_DL_. The correlation coefficient between ADC and SUV values with a 60% ROI mask was ρ = −0.51 for ADC_Kurtosis_ and SUV_Skewness_, confirming the statistically significant difference between PET_DL_ and PET_MRI_ values (Figure 4).

The correlations between ADC_Kurtosis_ and SUV_Skewness_ for each method were as follows: ρ = −0.51 for PET_CT_, ρ = −0.47 for PET_MRI,_ and ρ = −0.49 for PET_DL_. The correlation coefficients between ADC_Kurtosis_ and SUV_Kurtosis_ were as follows: ρ = −0.46 for PET_CT_, ρ = −0.47 for PET_MRI,_ and ρ = −0.48 for PET_DL_. As for the ADC_mean_, ADC_min_, ADC_median_ and SUV_mean_, SUV_max_, SUV_min_, and SUV_median_ parameters, the correlation coefficient became more pronounced, though no significant difference between the methods was observed. The results of SUV and ADC values thresholded by 10%, 20%, 30%, 40%, and 50% are presented in the Supplementary Material.

The ADC and SUV values, together with an average relative absolute error (%) for PET_MRI_ and PET_DL_ images for the ROI of cancer in peripheral and transitional zones for 27 subjects, are shown in Table 1. The difference between PET_MRI_ and PET_DL_ images was statistically significant for SUV_max,_ SUV_kurtosis,_ and SUV_Skewness_, as assessed using the t-test with *p* < 0.05. The mean errors for the PET_MRI_ images were as follows: 2.7%, 2.6%, 29.0%, and 12.6%; for the PET_DL_ images, the errors were as follows: 1.5%, 1.4%, 9.1%, and 2.1%, for mean, max, kurtosis and skewness values, respectively.

## 3. Discussion

This study demonstrated the potential of DL-derived SUV values to enhance the correlation with ADC compared to conventional PET-MR methods, offering a novel approach to prostate cancer imaging. Histopathological validation remains the gold standard for prostate cancer diagnosis and grading. While our study focused on imaging correlations, future research should incorporate systematic histopathological comparison to confirm the accuracy of ADC and SUV correlations. This would strengthen the clinical applicability of our findings and address potential limitations in imaging-based assessments.

In the diagnosis of prostate cancer, the false-negative rate of biopsies ranges from approximately 15% to 35% [34]. MRI techniques improve the detection rate of prostate cancer, while also avoiding unnecessary invasive biopsies. Furthermore, a recent study shows that the histopathological features of prostate cancer may be assessed using ADC maps instead of biopsy [35] since ADC maps reflect the degree of diffusion of water molecules in tissues. However, ^68^Ga-PSMA-11 PET is even more effective in detecting primary lesions in prostate cancer than MRI. The appearance of the hybrid PET-MR systems has made it possible to combine PET and DWI imaging, which, due to its high accuracy, is often the method of choice for detecting prostate cancer [21,36]. The present study aimed to investigate the correlation between DL-based techniques and multimodal imaging methods for prostate cancer lesions in the PZ and TZ regions by using SUV and ADC parameters and thresholding the ROI mask.

The inverse correlation between ADC and SUV values was observed using histogram analyses for PET and DWI images. In this study, PET images were derived using DL techniques and multimodal imaging methods. The corresponding ADC and SUV values were inversely correlated, confirming the results described by other studies [23,37], since prostate cancer is associated with lower ADC and higher SUV values [38,39]. Several studies have verified that ^68^Ga-PSMA-11 PET-MRI can improve the detection rate of prostatic cancer, especially in the PZ and TZ. ADC integration with ^68^Ga-PSMA-11 PET/MRI provides some insights into the tumor behavior and aggressiveness. Furthermore, ADC values can be used as a biomarker for therapeutic responses and predictions during treatment [40]. Our work aims not merely to present the repetition of existing results in the literature, but to demonstrate a new perspective on the correlation between ADC and SUV values by using CT, MRI, and DL AC maps in reconstructing PET-MR images.

Numerous compelling comparative analyses of PET and DWI have already been presented for hybrid PET/MRI systems [41,42]. Buchbender et al. showed that the simultaneous acquisition of FDG PET and DWI in PET/MRI systems does not affect ADC measurements or signal-to-noise ratios for tumors [41]. Hambrock et al. have shown the negative correlations between ADC and the Gleason score in the PZ in prostate cancer and demonstrated that ADC can differentiate between low-, intermediate- and high-grade tumors [43]. Reischauer et al. presented DWI analyses of bone metastases in prostate cancer, where the mean ADC values of bone metastases increased significantly during antiandrogen therapy [44]. These studies prove that the combination of PET and DWI imaging may be a promising tool in prostate cancer detection.

Histopathological validation remains the gold standard for prostate cancer diagnosis and grading. While our study focused on imaging correlations, future research should incorporate systematic histopathological comparison to confirm the accuracy of ADC and SUV correlations. This would strengthen the clinical applicability of our findings and address potential limitations in imaging-based assessments.

A limitation of this study was the absence of a control group without prostate cancer. Including such a group would have offered a stronger comparative framework for assessing ADC and SUV values. Nevertheless, the primary objective of this study was to explore the correlation between imaging parameters in confirmed cases of prostate cancer.

The small sample size of 32 patients, with only 27 remaining in the final analysis after excluding five due to issues with the registration of ADC maps to T2-weighted images, limited the ability to stratify patients according to cancer severity. To validate these findings, larger prospective studies are necessary to assess the prognostic value of combining SUV and ADC analyses. Furthermore, incorporating control groups would improve diagnostic accuracy and enhance the generalizability of the results across diverse populations.

There is a slight difference in correlations between PET_MRI_ and PET_DL_ images. However, we expect that larger patient groups would likely have resulted in more prominent differences in correlations for PET_MRI_ and PET_DL_ images which should improve the DL model creating attenuation correction maps. The correlation results between SUV and ADC parameters in prostate cancer are encouraging. Further work needs to be undertaken and evaluated regarding parameters that can usefully serve the evaluation of the biological prognosis of tumors, and which can lead to more positive and impactful treatment strategies. Finally, for further evaluation of the values of SUV and ADC, a large-scale prospective study with prognostic follow-up findings of the simultaneous ^68^Ga-PSMA-11 PET/MRI is needed.

## 4. Materials and Methods

### 4.1. Patients and Methods

The PET-CT and PET-MR studies were performed in accordance with ethical guidelines. Ethical approval was obtained from the Human Research Ethics Committee (HREC) of Monash University, approval number 0780. All participants provided written informed consent after receiving a detailed explanation of the study’s purpose and procedures. A total of 32 male patients were diagnosed with prostate cancer (mean age: 65 ± 8 years; range: 45–78 years), of which 27 were included in the final analysis after exclusions. All 32 patients included in the study underwent imaging before any biopsy procedures to ensure that ADC and SUV values were unaffected by biopsy-related changes in tissue characteristics. Data from all patients were grouped using Gleason scores: group 2: 3 + 4 = 7 (*n* = 6 patients, 18.8%); group 3: 4 + 3 = 7 (*n* = 13 patients, 40.6%); group 4: 4 + 4 = 8 (*n* = 6 patients, 18.8%); group 5: 4 + 5 = 9 (*n* = 7 patients, 21.8%).

### 4.2. Study Protocol

PET-CT data were acquired using a GE Discovery 710 PET-CT scanner (GE Healthcare, Waukesha, WI, USA) with a pixel size of 1.367 mm × 1.367 mm, matrix of 512 × 512, slice thickness of 3.2 mm, an operating energy range of 120–140 kV and a tube current of 150 mA. The average administered dose of ^68^Ga-PSMA was 174 ± 26 MBq (range 115–250 MBq).

The PET-MR image acquisition was performed approximately 60 min after the PET-CT scans using a Biograph mMR PET_MR scanner (Siemens Healthineers, Erlangen, Germany) Imaging parameters were as follows: Dixon images with a voxel size of 2.604 × 2.604 × 3.12 mm^3^; a matrix of 192 × 120; repetition time of 3.96 ms; first echo time of 1.23 ms; flip angle of 9°; and 128 slices. A thirty-minute PET list-mode dataset was acquired, and the images were reconstructed on the scanner console. DWI images were obtained in the axial plane, with the b-values of 0 and 800 s/mm^2^, using the following parameters: repetition time/echo time = 4300 m/69 ms, a matrix of 260 × 260, slice thickness of 3.6 mm, image resolution of 0.962 × 0.962 mm^2^, flip angle of 90°, and 78 slices. T_2_-weighted images were also acquired at the end of the examination: a voxel size of 0.804 × 0.804 × 5.2 mm^3^, a matrix of 448 × 448, repetition time/echo time = 4000 m/95 ms, flip angle of 140°, and 40 slices.

PET image reconstructions were performed using the following μ-maps: reference co-registered CT μ-map (μ-map_CT_); Dixon μ-map with bone information (μ-map_MRI_); and a DL-based μ-map (μ-map_DL_). Reference AC μ-maps were reconstructed using a 3T PET-MR scanner (Siemens 3T Biograph mMR, Monash Biomedical Imaging, Clayton, Australia). Forty-minute PET list-mode data were acquired and reconstructed using the following parameters: point spread function (PSF) algorithm; 3 iterations; 21 groups; 172 × 172 matrix and 127 slices; 4.173 × 4.173 × 2.032 mm^3^ image resolution; AC and absolute scatter correction; the isotope gamma half-life of 4057.7 (sec); the isotope branching factor of 0.89. The reconstructed PET images were smoothed using a 3 mm Gaussian filter and denoted as PET_ct_, PET_MRI_, and PET_DL_.

### 4.3. Deep Learning-Based AC μ-Maps

Generative adversarial networks, as described in our prior investigational endeavors, were employed for developing a deep learning model designed to predict DL-based AC μ-maps [45]. The input to the model comprised Dixon images, while the corresponding co-registered CT μ-maps served as the output. The DL network architecture consisted of a generator and a discriminator [46]. The generator adopted a U-Net architecture [47], and the discriminator was a convolutional neural network structured with seven convolution layers. The dataset incorporated data from 18 patients, resulting in a total of 2160 slices. This dataset was augmented to 270,000 slices for training the GANs. Augmentation involved the deformation fields ranging from 0.94 to 1.06, resulting in 125 augmented deformation fields for each co-registered dataset [45,48]. Subsequently, registered CT images underwent conversion into continuous linear attenuation coefficients at 511 keV [49]. In total, 18 patients were co-registered, generating 2250 datasets for training the deep learning model. Diverging from the prior methodology, the workflow was refined by incorporating K-fold cross-validations, effectively dividing the dataset into training and test sets. This procedural adaptation contributes to the heightened robustness and reliability of the deep learning model. K-fold cross-validation ensures the inclusion of all datasets for both testing and training, guaranteeing each data point’s presence in the test set precisely once and in the training set K-1 times [50]. In each iteration of the DL network, 24 subjects were designated for training and 8 for testing. This process was iterated four times, resulting in the creation of distinct DL models. The training of the DL networks was performed using the following parameters: Nadam optimizer, a learning rate of 0.0002, 270,000 iterations, and 50 epochs. The MASSIVE computer cluster was used for the computational training of the model [51].

### 4.4. SUV and ADC Histogram Analyses

The reference/co-registered CT μ-maps, as well as MR and DL-based AC μ-maps were used to reconstruct PET images. Reconstructed PET images and ADC maps were co-registered with T2-weighted images using the non-linear transformation from Advanced Normalization Tools [48]. Regions of interest (ROIs) for prostate cancer lesions in the peripheral and transitional zones were manually delineated on co-registered T2-weighted images by two experienced readers with collectively more than 10 years of experience in prostate imaging. Both radiologists independently assessed the images, and discrepancies were resolved through consensus to ensure accurate ROI delineation in MR image axial slices. ROIs were created by mapping the regions on each 2D slice of a 3D volume using MRIcron software (www.mccauslandcenter.sc.edu/mricro/mricron) (accessed on 10 January 2022). From the reconstructed PET images, the SUV values were computed to assess patient responses to therapy [52]. The following PET parameters were calculated: mean SUV (SUV_mean_); maximum SUV (SUV_max_); minimum SUV (SUV_min_); and median SUV (SUV_median_). ROI analyses of the PZ and TZ zones in prostate cancer were used to calculate the corresponding PET parameters. For the ADC maps, the following parameters were calculated: mean ADC (ADC_mean_); maximum ADC (ADC_max_); minimum ADC (ADC_min_); and median ADC (ADC_median_). Histogram characteristics were calculated based on kurtosis and skewness values [53]. The manually created ROIs masking cancer in the PZ and TZ from T2-weighted MR axial slices were used to calculate SUV_max_ values for each patient. For the optimal SUV threshold for maximum correlation with ADC values, six different ROI masks of cancer were created by applying a percentage threshold of SUV_max_ values (viz. 10%, 20%, 30%, 40%, 50%, 60%). Using each mask, PET and ADC parameters were calculated. The correlations between these values were later investigated. We also calculated the average relative absolute error of SUV values between the referenced PETct, PET_MRI_, and PET_DL_ images. The average relative errors of the SUV values between the referenced PET_CT_, PET_MRI_, and PET_DL_ images were computed using the following formula:

$${avg.RAE}_{x}=\frac{1}{N}\cdot \sum_{i\in ROI}\frac{{|P}_{x}-P_{ref}|}{P_{ref}}\cdot 100,$$

where N is the total of pixels within the ROI, P_x_ are the reconstructed PET images (PET_MRI_, and PET_DL_) and PET_ref_ is the reference images PET_CT_.

### 4.5. Statistical Testing

The ADC and SUV values were analyzed using descriptive statistics, mean and standard deviation. Mann–Whitney–Wilcoxon’s nonparametric signed two-tailed rank test was used to detect any statistically significant differences between ADC and SUV values. The difference between ADC and SUV values was considered significant for *p* < 0.05. Spearman’s correlation coefficient (ρ) was calculated to observe the relationship between ADC and SUV.

## 5. Conclusions

The combination of SUV and ADC values can serve as a valuable parameter for the diagnosis of prostate cancer and the evaluation of the biological behavior of the tumor. This study showed a strong inverse correlation between ADC and SUV values in PZ and TZ of prostate cancer. There is also a slight difference between PETMRI and PETDL methods in TZ and PZ, but this is not statistically significant. Our study was based on data from only 27 patients. This makes it impossible to divide the group into varying (or substantive) cancer severity categories. In future works, we plan to increase the group of patients to enable research on different cancer severity classes.

## Figures and Tables

**Table 1:** ADC and SUV values for reconstructed PET_ct_ images and average relative absolute error (%) for PET_MRI_ and PET_DL_ images for the ROI of cancer in PZ and TZ for twenty-seven subjects.

Parameters	Cancer in PZ and TZ				
	ADC Values (×10^−3^ mm^2^ s^−1^)	PET_CT_ (SUV)	Average Relative Absolute Error (%)		
			PET_MRI_	PET_DL_	*t*-Test
Mean	1.0 ± 0.1	9.9 ± 1.5	2.7 ± 0.5	1.5 ± 0.2	0.08
Max	1.7 ± 0.1	15.6 ± 2.7	2.6 ± 0.5	1.4 ± 0.2	0.04
Min	0.5 ± 0.1	6.7 ± 0.9	3.6 ± 1.1	1.5 ± 0.2	0.06
Median	1.0 ± 0.1	9.6 ± 1.4	2.2 ± 0.4	1.5 ± 0.2	0.11
Kurtosis	0.2 ± 0.4	0.2 ± 0.6	29.0 ± 7.7	9.1 ± 2.7	0.01
Skewness	0.4 ± 0.1	0.6 ± 0.1	12.6 ± 4.7	2.1 ± 0.5	0.03

## Data Availability

The datasets used and/or analyzed materials for the current study are available, on reasonable request, from the corresponding author.

## Supplementary Materials

The following supporting information can be downloaded at: https://www.mdpi.com/article/10.3390/ijms26030905/s1.

## Abbreviations

AC	attenuation correction
ADC	apparent diffusion coefficient
DL	deep learning
DWA	diffusion-weighted imaging
GAN	generative adversarial network
LN	lymph node
PET-CT	positron emission tomography—computed tomography
PET-MR	positron emission tomography—magnetic resonance imaging
PSA	prostate-specific antigen
PSMA	prostate-specific membrane antigen
PZ	peripheral zone
ROI	region of interest
SUV	standardized uptake values
TZ	transition zone
