# Supplementary material for: Correlations Between MR Apparent Diffusion Coefficients and PET Standard Uptake Values in Simultaneous MR-PET Imaging of Prostate Cancer
Source: Int J Mol Sci. 2025 Jan 22;26(3):905. doi: 10.3390/ijms26030905 (PMC11817574; doi:10.3390/ijms26030905)
Supplement: Supplementary file 1 [file ijms-26-00905-s001.zip › ijms-3405113-supplementary.pdf]

Supporting Information:

Correlations between MR apparent diffusion coefficients and PET standard uptake values in simultaneous MR-PET imaging of prostate cancer

Andrii Pozaruk[1,2,7], Vitaliy Atamaniuk[2], Kamlesh Pawar[3,7], Alexandra Carey[5,7], Jeremy Cheng[6], Marian Cholewa[2], Jeremy Grummet[6], Zhaolin Chen[4,7], Gary Egan[3,7]

[1]Institute of Medical Sciences - Department of Photomedicine and Physical Chemistry, The Medical College of The University of Rzeszów, Rzeszów, Poland;

[2]Institute of Physics, College of Natural Sciences, University of Rzeszów, Poland

[3]Monash Institute of Cognitive and Clinical Neurosciences and School of Psychological Sciences, Monash University, Clayton, Australia

[4]Department of Data Science and AI, Faculty of Information Technology, Monash University, Clayton, Australia

[5]Monash Imaging, Monash Health, Clayton, Australia

[6]Department of Surgery, Central Clinical School, Monash University, Melbourne, Australia

[7]Monash Biomedical Imaging, Monash University, Clayton, Australia;

Email: apozaruk@ur.edu.pl

Table S1: SUV values for the reconstructed PET_{CT}, PET_{MRI} and PET_{DL} images, and ADC values for the twenty-seven patients using ROI threshold of 10% for cancer in PZ and TZ.

Patients	Group	Gleason grade	Prostate cancer in PZ and TZ											
			Reconstructed PET images (SUV)									ADC map ($\times 10^{-3}$ mm^2 s^{-1})		
			CT_{mean}	CT_{max}	CT_{min}	MR_{mean}	MR_{max}	MR_{min}	DL_{mean}	DL_{max}	DL_{min}	ADC_{mean}	ADC_{max}	ADC_{min}
1	2	3+4=7	4.13	4.70	3.70	4.16	4.99	3.71	4.06	4.63	3.63	0.79	1.35	0.22
2			6.10	9.14	3.96	5.79	8.67	3.75	5.81	8.68	3.77	0.95	1.55	0.64
3			2.58	2.68	2.43	2.58	2.70	2.41	2.52	2.62	2.37	0.89	1.44	0.67
4			5.92	7.63	4.97	5.98	7.66	5.02	5.90	7.60	4.93	1.16	1.61	0.78
5			8.17	17.47	4.37	8.56	16.90	4.24	8.65	17.26	4.31	1.35	2.06	0.45
6			16.97	31.24	7.79	16.65	30.42	7.64	16.59	30.45	7.63	1.00	1.61	0.44
7	3	4+3=7	10.63	14.54	7.83	11.01	14.91	8.12	10.58	14.47	7.80	0.90	1.63	0.21
8			8.92	11.53	6.74	8.69	11.18	6.60	8.97	11.59	6.77	1.43	1.86	0.95
9			13.78	24.42	8.96	13.68	24.64	8.78	13.54	23.92	8.84	0.97	1.70	0.49
10			22.87	56.46	10.73	21.82	53.62	10.14	22.23	54.85	10.43	1.02	1.86	0.30
11			9.21	13.48	6.44	8.95	13.07	6.24	9.05	13.24	6.32	0.99	1.39	0.76
12			9.85	12.76	7.82	9.71	12.67	7.62	9.54	12.39	7.58	1.01	1.56	0.21
13			8.35	11.92	6.66	8.40	12.05	6.62	8.40	12.01	6.69	1.28	2.05	0.81
14			13.87	22.95	11.03	14.02	22.95	11.08	13.96	23.09	11.11	0.86	1.62	0.44
15			8.53	12.86	5.57	8.03	12.05	5.21	8.50	12.79	5.54	1.07	1.54	0.78
16			2.99	3.60	2.69	2.98	3.65	2.65	2.93	3.55	2.63	1.23	1.93	0.64
17			3.41	3.82	3.02	3.41	3.82	2.95	3.36	3.78	2.99	1.13	1.65	0.76
18	4	4+4=8	4.44	7.03	2.19	4.30	6.92	1.53	4.49	7.05	2.17	0.83	1.95	0.38
19			7.68	13.44	5.58	7.71	13.25	5.56	7.67	13.36	5.59	1.03	1.60	0.53
20			4.67	5.42	4.22	4.52	5.27	4.00	4.55	5.29	4.08	0.99	1.58	0.49
21			4.69	5.93	3.90	4.82	6.05	3.94	4.70	5.94	3.91	1.01	1.52	0.55
22	5	4+5=9	6.50	8.34	5.66	6.38	8.11	5.53	6.53	8.37	5.69	1.08	1.66	0.52
23			4.16	4.83	3.55	4.50	5.44	3.84	4.14	4.81	3.52	1.05	1.70	0.05
24			16.75	25.32	8.93	16.79	25.32	9.20	16.89	25.55	8.97	0.66	1.56	0.31
25			8.90	13.06	6.76	8.65	12.74	6.57	8.75	12.89	6.69	0.80	1.34	0.44
26			11.55	18.58	6.29	11.66	2.93	6.32	11.32	18.23	6.12	1.24	2.00	0.61
27			40.75	59.49	27.28	38.98	56.99	26.48	40.06	58.42	26.79	1.15	2.04	0.84
mean			9.86	15.65	6.63	9.73	14.77	6.50	9.76	15.43	6.55	1.03	1.68	0.52
SEM			1.51	2.73	0.91	1.44	2.65	0.89	1.48	2.67	0.90	0.03	0.04	0.04

Table S2: SUV values for the reconstructed PET_{CT}, PET_{MRI} and PET_{DL} images, and ADC values for the twenty-seven patients using the thresholding ROI by 10% for cancer in PZ and TZ.

Patients	Group	Gleason grade	Prostate cancer in PZ and TZ											
			Reconstructed PET images (SUV)									ADC map ($\times 10^{-3}$ mm^2 s^{-1})		
			CT_{med}	CT_{Kur}	CT_{Ske}	MR_{med}	MR_{Kur}	MR_{Ske}	DL_{med}	DL_{Kur}	DL_{Ske}	ADC_{med}	ADC_{Kur}	ADC_{Ske}
1	2	3+4=7	4.10	0.79	0.75	4.12	7.29	1.04	4.02	0.65	0.71	0.79	0.44	0.08
2			5.83	-0.63	0.59	5.54	-0.66	0.57	5.54	-0.63	0.59	0.91	-0.04	0.67
3			2.59	-0.15	-0.73	2.58	-0.02	-0.61	2.53	-0.07	-0.79	0.87	1.62	1.06
4			5.85	0.38	0.71	5.92	0.49	0.74	5.83	0.27	0.66	1.16	-0.51	0.11
5			8.40	2.10	1.20	8.30	2.00	1.13	8.35	2.03	1.18	1.41	-0.57	-0.47
6			15.45	-0.76	0.59	15.19	-0.75	0.59	15.11	-0.76	0.59	0.96	0.08	0.40
7	3	4+3=7	10.49	-0.69	0.35	10.88	-0.71	0.33	10.40	-0.68	0.35	0.89	0.92	0.32
8			8.69	-0.99	0.37	8.46	-0.96	0.38	8.74	-0.99	0.37	1.44	-0.25	-0.40
9			13.07	0.20	0.90	12.96	0.27	0.92	12.84	0.18	0.89	0.93	0.25	0.72
10			20.06	0.21	0.93	19.23	0.18	0.92	19.51	0.20	0.93	1.00	0.11	0.48
11			8.80	-0.51	0.65	8.57	-0.54	0.63	8.66	-0.51	0.65	0.97	1.99	1.16
12			9.52	-0.79	0.51	9.41	-0.84	0.47	9.21	-0.82	0.49	1.05	-0.18	-0.47
13			8.16	0.88	1.10	8.18	0.88	1.09	8.21	0.91	1.12	1.27	-0.08	0.41
14			13.02	2.17	1.65	13.23	2.18	1.64	13.10	2.17	1.65	0.84	0.86	0.89
15			8.26	-0.38	0.60	7.79	-0.41	0.58	8.24	-0.38	0.60	1.04	-0.03	0.77
16			2.95	0.70	0.96	2.93	0.57	0.90	2.90	0.71	0.96	1.21	-0.63	0.26
17			3.38	-0.57	0.37	3.40	-0.25	-0.02	3.34	-0.54	0.39	1.10	0.09	0.66
18	4	4+4=8	4.44	0.89	-0.45	4.34	1.07	-0.80	4.51	0.82	-0.47	0.78	2.17	1.35
19			7.59	2.60	1.13	7.71	2.18	0.92	7.59	2.50	1.08	0.99	-0.09	0.54
20			4.62	-0.07	0.76	4.47	0.03	0.75	4.49	-0.04	0.78	1.00	-0.99	-0.05
21			4.58	0.27	0.88	4.74	0.19	0.73	4.59	0.26	0.84	1.02	-1.04	0.14
22	5	4+5=9	6.38	0.04	0.84	6.25	0.00	0.78	6.41	0.05	0.84	1.08	-0.34	0.21
23			4.14	-0.39	0.34	4.47	-0.90	0.32	4.10	-0.34	0.35	1.02	0.11	-0.53
24			16.23	-0.57	0.47	16.24	-0.58	0.47	16.36	-0.57	0.47	0.59	1.33	1.30
25			8.80	0.98	0.87	8.56	0.98	0.89	8.65	1.02	0.90	0.78	0.06	0.63
26			10.95	-0.77	0.54	11.06	-0.78	0.53	10.73	-0.76	0.54	1.26	-0.88	0.18
27			39.69	-0.57	0.54	37.88	-0.55	0.57	39.11	-0.57	0.54	1.05	0.37	1.11
mean			9.48	0.16	0.64	9.34	0.38	2.08	9.37	1.40	0.63	1.01	0.17	0.42
SEM			1.43	0.18	0.08	1.36	0.32	1.48	1.40	0.15	0.09	0.03	0.16	0.10

Table S3: SUV values for the reconstructed PET_{CT}, PET_{MRI} and PET_{DL} images, and ADC values for the twenty-seven patients using ROI threshold of 20% for cancer in PZ and TZ.

Patients	Group	Gleason grade	Prostate cancer in PZ and TZ											
			Reconstructed PET images (SUV)									ADC map ($\times 10^{-3}$ mm^2 s^{-1})		
			CT_{mean}	CT_{max}	CT_{min}	MR_{mean}	MR_{max}	MR_{min}	DL_{mean}	DL_{max}	DL_{min}	ADC_{mean}	ADC_{max}	ADC_{min}
1	2	3+4=7	4.13	4.70	3.70	4.16	4.99	3.71	4.06	4.63	3.63	0.79	1.35	0.22
2			6.10	9.14	3.96	5.79	8.67	3.75	5.81	8.68	3.77	0.95	1.55	0.64
3			2.58	2.68	2.43	2.58	2.70	2.41	2.52	2.62	2.37	0.89	1.44	0.67
4			5.92	7.63	4.97	5.92	7.66	5.02	5.90	7.60	4.93	1.16	1.61	0.78
5			8.71	17.47	4.37	8.56	16.90	4.24	8.65	17.26	4.31	1.35	2.06	0.45
6			16.97	31.24	7.79	16.65	30.42	7.64	16.59	30.45	7.63	1.00	1.61	0.44
7	3	4+3=7	10.63	14.54	7.83	11.01	14.91	8.12	1.58	14.47	7.80	0.90	1.63	0.21
8			8.92	11.53	6.71	8.69	11.18	6.60	8.97	11.59	6.77	1.43	1.86	0.95
9			13.78	24.42	8.96	13.68	24.64	8.78	13.54	23.92	8.84	0.97	1.70	0.49
10			22.97	56.46	11.29	21.92	53.62	10.74	22.33	54.85	10.97	1.02	1.85	0.30
11			9.21	13.48	6.44	8.95	13.07	6.24	9.05	13.24	6.32	0.99	1.39	0.76
12			9.85	12.76	7.82	9.71	12.67	7.62	9.54	12.39	7.58	1.01	1.56	0.21
13			8.35	11.93	6.66	8.40	12.05	6.62	8.40	12.01	6.69	1.28	2.05	0.81
14			13.87	22.95	11.03	14.02	22.95	11.08	13.96	23.09	11.11	0.84	1.62	0.44
15			8.53	12.86	5.57	8.03	12.05	5.21	8.50	12.79	5.54	1.07	1.54	0.78
16			2.99	3.60	2.69	2.98	3.56	2.65	2.93	3.55	2.63	1.23	1.93	0.64
17			3.41	3.82	3.02	3.41	3.82	2.95	3.36	3.78	2.99	1.13	1.65	0.76
18	4	4+4=8	4.44	7.03	2.19	4.30	6.92	1.53	4.49	7.05	2.17	0.83	1.95	0.38
19			7.68	13.44	5.58	7.71	13.25	5.56	7.67	13.36	5.55	1.03	1.60	0.53
20			4.67	5.42	4.22	4.52	5.27	4.00	4.55	5.29	4.08	0.99	1.58	0.49
21			4.69	5.93	3.90	4.82	6.05	3.94	4.70	5.94	3.91	1.01	1.52	0.55
22	5	4+5=9	6.50	8.34	5.66	6.38	8.11	5.53	6.53	8.37	5.69	1.08	1.66	0.52
23			4.14	4.83	3.55	4.50	5.44	3.84	4.14	4.81	3.52	1.05	1.70	0.05
24			13.75	25.32	8.93	16.79	25.32	9.20	16.89	25.55	8.97	0.66	1.56	0.31
25			8.90	13.06	6.76	8.65	12.74	6.57	8.75	12.89	6.69	0.80	1.34	0.44
26			11.55	18.58	6.29	11.66	18.78	6.32	11.32	18.23	6.12	1.24	2.00	0.61
27			40.75	59.49	27.28	38.98	56.99	26.48	40.06	58.42	26.79	1.15	2.04	0.84
mean			9.40	15.65	6.65	9.73	15.36	6.53	9.43	15.43	6.56	1.03	1.67	0.52
SEM			1.50	2.73	0.92	1.44	2.61	0.90	1.51	2.67	0.90	0.03	0.04	0.04

Table S4: SUV values for the reconstructed PET_{CT}, PET_{MRI} and PET_{DL} images, and ADC values for the twenty-seven patients using ROI threshold of 20% for cancer in PZ and TZ.

Patients	Group	Gleason grade	Prostate cancer in PZ and TZ											
			Reconstructed PET images (SUV)									ADC map ($\times10^{-3}$ mm^2 s^{-1})		
			CT_{med}	CT_{Kur}	CT_{Ske}	MR_{med}	MR_{Kur}	MR_{Ske}	DL_{med}	DL_{Kur}	DL_{Ske}	ADC_{med}	ADC_{Kur}	ADC_{Ske}
1	2	3+4=7	4.10	0.79	0.75	4.12	1.29	1.04	4.02	0.65	0.71	0.79	0.44	0.08
2			5.83	-0.63	0.59	5.54	-0.66	0.57	5.54	-0.63	0.59	0.91	-0.04	0.67
3			2.59	-0.15	-0.73	2.58	-0.02	-0.61	2.53	-0.07	-0.79	0.87	1.62	1.06
4			5.85	0.38	0.71	5.92	0.49	0.74	5.83	0.27	0.66	1.16	-0.51	0.11
5			8.40	2.10	1.20	8.30	2.00	1.13	8.35	2.03	1.18	1.41	-0.57	-0.47
6			15.45	-0.76	0.59	15.19	-0.75	0.59	15.11	-0.76	0.59	0.96	0.08	0.40
7	3	4+3=7	10.49	-0.69	0.35	10.88	-0.71	0.33	10.40	-0.68	0.35	0.89	0.92	0.32
8			8.69	-0.99	0.37	8.46	-0.96	0.38	8.74	-0.99	0.37	1.44	-0.25	-0.40
9			13.07	0.20	0.90	12.96	0.27	0.92	12.84	0.18	0.89	0.93	0.25	0.72
10			20.24	0.20	0.93	19.45	0.17	0.92	19.71	0.20	0.93	1.00	0.09	0.48
11			8.80	-0.51	0.65	8.57	-0.54	0.63	8.66	-0.51	0.65	0.97	1.99	1.16
12			9.52	-0.79	0.51	9.341	-0.84	0.47	9.21	-0.82	0.49	1.05	-0.18	-0.47
13			8.16	0.88	1.10	8.18	0.88	1.09	8.21	0.91	1.12	1.27	-0.08	0.41
14			13.02	2.17	1.65	13.23	2.18	1.64	13.10	2.17	1.65	0.84	0.86	0.89
15			8.26	-0.38	0.60	7.79	-041	0.58	8.24	-0.38	0.60	1.04	-0.03	0.77
16			2.95	0.70	0.96	2.93	0.57	0.90	2.90	0.71	0.96	1.21	-0.63	0.26
17			3.38	-0.57	0.37	3.40	-0.25	-0.02	3.34	-0.54	0.39	1.10	0.09	0.66
18	4	4+4=8	4.44	0.89	-0.45	4.34	1.07	-0.80	4.51	0.82	-0.47	0.78	2.17	1.35
19			7.59	2.60	1.13	7.71	2.18	0.92	7.59	2.50	1.08	0.99	-0.09	0.54
20			4.62	-0.07	0.76	4.47	0.03	0.75	4.49	-0.04	0.78	1.00	-0.99	-0.05
21			4.58	0.27	0.88	4.74	0.19	0.73	4.59	0.26	0.84	1.02	-1.04	0.14
22	5	4+5=9	6.38	0.04	0.84	6.25	0.00	0.78	6.41	0.05	0.84	1.08	-0.34	0.21
23			4.14	-0.39	0.34	4.47	-0.90	0.32	4.10	-0.34	0.35	1.02	0.11	-0.53
24			16.23	-0.57	0.47	16.24	-0.58	0.47	16.36	-0.57	0.47	0.59	1.33	1.30
25			8.80	0.98	0.87	8.56	0.98	0.89	8.65	1.02	0.90	0.78	0.06	0.63
26			10.95	-0.77	0.54	11.06	-0.78	0.53	10.73	-0.76	0.54	1.24	-0.88	0.18
27			39.69	-0.57	0.54	37.88	-0.55	0.57	39.11	-0.57	0.54	1.05	0.37	1.11
mean			9.48	0.16	0.64	9.72	0.16	0.60	9.38	0.15	0.63	1.01	0.17	0.42
SEM			1.43	0.18	0.08	1.40	0.18	0.09	1.41	0.18	0.09	0.03	0.16	0.10

Table S5: SUV values for the reconstructed PET_{CT}, PET_{MRI} and PET_{DL} images, and ADC values for the twenty-seven patients using ROI threshold of 30% for cancer in PZ and TZ.

Patients	Group	Gleason grade	Prostate cancer in PZ and TZ											
			Reconstructed PET images (SUV)									ADC map ($\times 10^{-3}$ $mm^2 s^{-1}$)		
			CT_{mean}	CT_{max}	CT_{min}	MR_{mean}	MR_{max}	MR_{min}	DL_{mean}	DL_{max}	DL_{min}	ADC_{mean}	ADC_{max}	ADC_{min}
1	2	3+4=7	4.13	4.70	3.70	4.16	4.99	3.71	4.06	4.63	3.63	0.79	1.35	0.22
2			6.10	9.14	3.96	5.49	8.67	3.75	5.81	8.68	3.77	0.95	1.55	0.64
3			2.58	2.68	2.43	2.58	2.70	2.41	2.52	2.62	2.37	0.89	1.44	0.67
4			5.92	7.63	4.97	5.98	7.66	5.02	5.90	7.60	4.93	1.16	1.61	0.78
5			8.74	17.47	5.27	8.59	16.90	5.12	8.68	17.26	5.20	1.35	2.06	0.45
6			17.14	31.24	9.43	16.76	30.42	9.14	16.72	30.45	9.17	1.00	1.61	0.44
7	3	4+3=7	10.63	14.54	7.83	11.01	14.91	8.12	10.49	14.47	7.80	0.90	1.63	0.21
8			8.92	11.53	6.74	8.69	11.18	6.60	8.97	11.59	6.77	1.43	1.86	0.95
9			13.78	24.42	8.96	13.68	24.64	8.78	13.54	23.92	8.84	0.97	1.70	0.49
10			27.94	56.46	16.93	26.57	53.62	16.11	27.15	54.85	16.47	0.95	1.68	0.30
11			9.21	13.48	6.44	8.95	13.07	6.24	9.05	13.24	6.32	0.99	1.39	0.76
12			9.85	12.76	7.82	9.71	12.67	7.62	9.54	12.39	7.58	1.01	1.56	0.21
13			8.35	11.92	6.66	8.40	12.05	6.62	8.40	12.01	6.69	1.28	2.05	0.81
14			13.87	22.95	11.03	14.02	22.95	11.08	13.96	23.09	11.11	0.86	1.62	0.44
15			8.53	12.86	5.57	8.03	12.05	5.21	8.50	12.79	5.54	1.07	1.54	0.78
16			2.99	3.60	2.60	2.98	3.56	2.65	2.93	3.55	2.63	1.21	1.93	0.64
17			3.41	3.82	3.02	3.41	3.82	2.95	3.36	3.78	2.99	1.13	1.65	0.76
18	4	4+4=8	4.44	7.03	2.19	4.53	6.92	2.08	4.49	7.05	2.17	0.83	1.95	0.38
19			7.68	13.44	5.58	7.71	13.25	5.56	7.67	13.36	5.55	1.03	1.60	0.53
20			4.67	5.42	4.22	4.52	5.27	4.00	4.55	5.29	4.08	0.99	1.58	0.49
21			4.69	5.93	3.90	4.82	6.05	3.94	4.70	5.94	3.91	1.01	1.52	0.55
22	5	4+5=9	6.50	8.34	5.66	6.38	8.11	5.53	6.53	8.37	5.69	1.08	1.66	0.52
23			4.16	4.83	3.55	4.50	5.44	3.84	4.14	4.81	3.52	1.05	1.70	0.05
24			16.75	25.32	8.93	16.79	25.32	9.20	16.89	25.55	8.97	0.66	1.56	0.31
25			8.90	13.06	6.76	8.67	12.74	6.57	8.75	12.89	6.69	0.80	1.34	0.44
26			11.55	18.58	6.29	11.66	18.78	6.32	11.32	18.23	6.12	1.24	2.00	0.61
27			40.75	59.49	27.28	38.98	56.99	26.48	40.06	58.42	26.79	1.15	2.04	0.84
mean			10.08	15.65	6.95	9.91	15.36	6.83	9.95	15.43	6.86	1.02	1.67	0.52
SEM			1.58	2.73	0.98	1.51	2.61	0.95	1.55	2.67	0.96	0.03	0.04	0.04

Table S6: SUV values for the reconstructed PET_{CT}, PET_{MRI} and PET_{DL} images, and ADC values for the twenty-seven patients using ROI threshold of 30% for cancer in PZ and TZ.

Patients	Group	Gleason grade	Prostate cancer in PZ and TZ											
			Reconstructed PET images (SUV)									ADC map ($\times10^{-3}$ mm^2 s^{-1})		
			CT_{med}	CT_{Kur}	CT_{Ske}	MR_{med}	MR_{Kur}	MR_{Ske}	DL_{med}	DL_{Kur}	DL_{Ske}	ADC_{med}	ADC_{Kur}	ADC_{Ske}
1	2	3+4=7	4.10	0.79	0.75	4.12	1.29	1.04	4.02	0.65	0.71	0.79	0.44	0.08
2			5.83	-0.63	0.59	5.54	-0.66	0.57	5.54	-0.63	0.59	0.91	-0.04	0.67
3			2.59	-0.15	-0.73	2.58	-0.02	-0.61	2.53	-0.07	-0.79	0.87	1.62	1.06
4			5.85	0.38	0.71	5.92	0.49	0.74	5.83	0.27	0.66	1.16	-0.51	0.11
5			8.42	2.16	1.25	8.31	2.06	1.19	8.36	2.10	1.24	1.41	-0.54	-0.48
6			15.64	-0.78	0.59	15.39	-0.76	0.60	15.25	-0.78	0.60	0.96	0.09	0.40
7	3	4+3=7	10.9	-0.69	0.35	10.88	-0.71	0.33	10.40	-0.68	0.35	0.89	0.92	0.32
8			8.69	-0.99	0.37	8.69	-0.96	0.38	8.74	-0.99	0.37	1.44	-0.25	-0.40
9			13.07	0.20	0.90	12.96	0.27	0.92	12.84	0.18	0.89	0.93	0.25	0.72
10			26.32	0.13	0.82	25.07	0.11	0.82	25.57	0.14	0.82	0.94	0.14	0.42
11			8.80	-0.51	0.65	8.57	-0.54	0.63	8.66	-0.51	0.65	0.97	1.99	1.16
12			9.52	-0.79	0.51	9.41	-084	0.47	9.21	-0.82	0.49	1.05	-0.18	-0.47
13			8.16	0.88	1.10	8.18	0.88	1.09	8.21	0.91	1.12	1.27	-0.08	0.41
14			13.02	2.17	1.65	13.23	2.18	1.64	13.10	2.17	1.65	0.84	0.86	0.89
15			8.26	-0.38	0.60	7.79	-0.41	0.58	8.24	-0.38	0.60	1.04	-0.03	0.77
16			2.95	0.70	0.96	2.93	0.57	0.90	2.90	0.71	0.96	1.21	-0.63	0.26
17			3.38	-0.57	0.37	3.40	-0.25	-0.02	3.34	-0.54	0.39	1.10	0.09	0.66
18	4	4+4=8	4.44	0.89	-0.45	4.42	0.74	0.38	4.51	0.82	-0.47	0.78	2.17	1.35
19			7.59	2.60	1.13	7.71	2.18	0.92	7.59	2.50	1.08	0.99	-0.09	0.54
20			4.62	-0.07	0.76	4.47	0.03	0.75	4.49	-0.04	0.78	1.00	-0.99	-0.05
21			4.58	0.27	0.88	4.74	0.19	0.73	4.59	0.26	0.84	1.02	-1.04	0.14
22	5	4+5=9	6.38	0.04	0.84	6.25	0.00	0.78	6.41	0.05	0.84	1.08	-0.34	0.21
23			4.14	-0.39	0.34	4.47	-0.90	0.32	4.10	-0.34	0.35	1.02	0.11	-0.53
24			16.23	-0.57	0.47	16.24	-0.58	0.47	16.36	-0.57	0.47	0.59	1.33	1.30
25			8.80	0.98	0.87	8.56	0.98	0.89	8.65	1.02	0.90	0.78	0.06	0.63
26			10.95	-0.77	0.54	11.06	-0.78	0.53	10.73	-0.76	0.54	1.26	-0.88	0.18
27			39.69	-0.57	0.54	37.88	-0.55	0.57	39.11	-0.57	0.54	1.05	0.37	1.11
mean			9.73	0.16	0.64	9.58	0.14	0.65	9.60	0.15	0.63	1.01	0.17	0.42
SEM			1.51	0.18	0.09	1.44	0.18	0.07	1.48	0.18	0.09	0.03	0.16	0.10

Table S7: SUV values for the reconstructed PET_{CT}, PET_{MRI} and PET_{DL} images, and ADC values for the twenty-seven patients using ROI threshold of 40% for cancer in PZ and TZ.

Patients	Group	Gleason grade	Prostate cancer in PZ and TZ											
			Reconstructed PET images (SUV)									ADC map ($\times10^{-3}$ mm^2 s^{-1})		
			CT_{mean}	CT_{max}	CT_{min}	MR_{mean}	MR_{max}	MR_{min}	DL_{mean}	DL_{max}	DL_{min}	ADC_{mean}	ADC_{max}	ADC_{min}
1	2	3+4=7	4.13	4.70	3.70	4.16	4.99	3.71	4.06	4.63	3.63	0.79	1.35	0.22
2			6.10	9.14	3.96	5.79	8.67	6.75	5.81	8.68	3.77	0.95	1.55	0.64
3			2.58	2.68	2.43	2.58	2.70	2.41	2.52	2.62	2.37	0.89	1.44	0.67
4			5.92	7.63	4.97	5.98	7.66	5.02	5.90	7.60	4.93	1.16	1.61	0.78
5			9.23	17.47	6.98	8.99	16.90	6.76	9.14	17.26	6.90	1.39	2.06	0.58
6			19.23	31.24	12.49	18.61	30.42	12.17	18.70	30.45	12.18	0.98	1.61	0.44
7	3	4+3=7	10.63	14.54	7.83	11.01	14.91	8.12	10.58	14.47	7.80	0.90	1.63	0.21
8			8.92	11.53	6.74	8.69	11.18	6.60	8.97	11.59	6.77	1.43	1.86	0.95
9			13.87	24.42	9.78	13.89	24.64	9.85	13.61	23.92	9.56	0.97	1.70	0.49
10			32.06	56.46	22.59	30.47	53.62	21.44	31.15	54.85	21.95	0.90	1.54	0.30
11			9.21	13.48	6.44	8.95	13.07	6.24	9.05	13.24	6.32	0.99	1.39	0.76
12			9.85	12.76	7.82	9.71	12.67	7.62	9.54	12.39	7.58	1.01	1.56	0.21
13			8.35	11.92	6.66	8.40	12.05	6.62	8.40	12.01	6.69	1.28	2.05	0.81
14			13.87	22.95	11.03	14.02	22.95	11.08	13.96	23.09	11.11	0.86	1.62	0.44
15			8.53	12.86	5.57	8.03	12.05	5.21	8.50	12.79	5.54	1.07	1.54	0.78
16			2.99	3.60	2.69	2.98	3.56	2.65	2.93	3.55	2.63	1.23	1.93	0.64
17			3.41	3.82	3.02	3.41	3.82	2.95	3.36	3.78	2.99	1.13	1.65	0.76
18	4	4+4=8	4.60	7.03	2.81	4.55	6.92	3.18	4.67	7.05	2.83	0.83	1.95	0.38
19			7.68	13.44	5.58	7.71	13.25	5.56	7.67	13.36	5.55	1.03	1.60	0.53
20			4.67	5.42	4.22	4.52	5.27	4.00	4.55	5.29	4.08	0.99	1.58	0.49
21			4.69	5.93	3.90	4.82	6.05	3.94	4.70	5.94	3.91	1.01	1.52	0.55
22	5	4+5=9	6.50	8.34	5.66	6.38	8.11	5.53	6.53	8.37	5.69	1.08	1.66	0.52
23			4.16	4.83	3.55	4.50	5.44	3.84	4.14	4.81	3.52	1.05	1.70	0.05
24			16.79	25.32	10.52	16.82	25.32	10.50	16.93	25.55	10.65	0.66	1.56	0.31
25			8.90	13.06	6.76	8.65	12.74	6.57	8.75	12.89	6.69	0.80	1.34	0.44
26			11.60	18.58	7.43	11.72	18.78	7.53	11.37	18.23	7.32	1.24	2.00	0.61
27			40.75	59.49	27.28	38.98	56.99	26.48	40.06	58.42	26.79	1.15	2.04	0.84
mean			10.34	15.65	7.49	10.16	15.36	7.49	10.20	15.43	7.39	1.02	1.66	0.53
SEM			1.66	2.73	1.09	1.59	2.61	1.04	1.63	2.67	1.07	0.03	0.04	0.04

Table S8: SUV values for the reconstructed PET_{CT}, PET_{MRI} and PET_{DL} images, and ADC values for the twenty-seven patients using ROI threshold of 40% for cancer in PZ and TZ.

Patients	Group	Gleason grade	Prostate cancer in PZ and TZ											
			Reconstructed PET images (SUV)									ADC map ($\times 10^{-3}$ $mm^2\,s^{-1}$)		
			CT_{med}	CT_{Kur}	CT_{Ske}	MR_{med}	MR_{Kur}	MR_{Ske}	DL_{med}	DL_{Kur}	DL_{Ske}	ADC_{med}	ADC_{Kur}	ADC_{Ske}
1	2	3+4=7	4.10	0.79	0.75	4.12	1.29	1.04	4.02	0.65	0.71	0.79	0.44	0.08
2			5.83	-0.63	0.59	5.54	-0.66	0.57	5.54	-0.63	0.59	0.91	-0.04	0.67
3			2.59	-0.15	-0.73	2.58	-0.02	-0.61	2.53	-0.07	-0.79	0.87	1.62	1.06
4			5.85	0.38	0.71	5.92	0.49	0.74	5.83	0.27	0.66	1.16	-0.51	0.11
5			8.73	2.80	1.59	8.55	2.75	1.55	8.64	2.72	1.56	1.43	-0.47	-0.45
6			18.23	-0.96	0.45	17.62	-0.94	0.48	17.67	-0.96	0.46	0.93	0.48	0.57
7	3	4+3=7	10.49	-0.69	0.35	10.88	-0.71	0.33	10.40	-0.68	0.35	0.89	0.92	0.32
8			8.69	-0.99	0.37	8.46	-0.96	0.38	8.74	-0.99	0.37	1.44	-0.25	-0.40
9			13.15	0.21	0.92	13.16	0.28	0.95	12.90	0.19	0.91	0.93	0.27	0.73
10			30.60	0.32	0.89	29.03	0.32	0.89	29.70	0.33	0.90	0.89	0.00	0.30
11			8.80	-0.51	0.65	8.57	-0.54	0.63	8.66	-0.51	0.65	0.97	1.99	1.16
12			9.52	-0.79	0.51	9.41	-0.84	0.47	9.21	-0.82	0.49	1.05	-0.18	-0.47
13			8.16	0.88	1.10	8.18	0.88	1.09	8.21	0.91	1.12	1.27	-0.08	0.41
14			13.02	2.17	1.65	13.23	2.18	1.64	13.10	2.17	1.65	0.84	0.86	0.89
15			8.53	-0.38	0.60	7.79	-0.41	0.58	8.24	-0.38	0.60	1.04	-0.03	0.77
16			2.95	0.70	0.96	2.93	0.57	0.90	2.90	0.71	0.96	1.21	-0.63	0.26
17			3.38	-0.57	0.37	3.40	-0.25	-0.02	3.34	-0.54	0.39	1.10	0.09	0.66
18	4	4+4=8	4.50	0.97	0.51	4.42	0.10	0.73	4.58	0.78	0.53	0.77	2.06	1.37
19			7.59	2.60	1.13	7.71	2.18	0.92	7.59	2.50	1.08	0.99	-0.09	0.54
20			4.62	-0.07	0.76	4.47	0.03	0.75	4.49	-0.04	0.78	1.00	-0.99	-0.05
21			4.58	0.27	0.88	4.74	0.19	0.73	4.59	0.26	0.84	1.02	-1.04	0.14
22	5	4+5=9	6.38	0.04	0.84	6.25	0.00	0.78	6.41	0.05	0.84	1.08	-0.34	0.21
23			4.14	-0.39	0.34	4.47	-0.90	0.32	4.10	-0.34	0.35	1.02	0.11	-0.53
24			16.25	-0.62	0.51	16.26	-0.63	0.51	16.40	-0.62	0.51	0.58	1.31	1.29
25			8.80	0.98	0.87	8.56	0.98	0.89	8.65	1.02	0.90	0.78	0.06	0.63
26			10.99	-0.78	0.55	11.09	-0.79	0.54	10.76	-0.77	0.55	1.26	-0.87	0.18
27			39.69	-0.57	0.54	37.88	-0.55	0.57	39.11	-0.57	0.54	1.05	0.37	1.11
mean			10.00	0.18	0.69	9.82	0.14	0.67	9.86	0.17	0.68	1.01	0.18	0.42
SEM			1.60	0.20	0.08	1.52	0.19	0.08	1.57	0.19	0.08	0.03	0.15	0.10

Table S9: SUV values for the reconstructed PET_{CT}, PET_{MRI} and PET_{DL} images, and ADC values for the twenty-seven patients using ROI threshold of 50% for cancer in PZ and TZ.

Patients	Group	Gleason grade	Prostate cancer in PZ and TZ											
			Reconstructed PET images (SUV)									DWI ($\times 10^{-3}$ mm^2 s^{-1})		
			CT_{mean}	CT_{max}	CT_{min}	MR_{mean}	MR_{max}	MR_{min}	DL_{mean}	DL_{max}	DL_{min}	ADC_{mean}	ADC_{max}	ADC_{min}
1	2	3+4=7	4.13	4.70	3.70	4.16	4.99	3.71	4.06	4.63	3.63	0.79	1.35	0.22
2			6.25	9.14	4.59	5.91	8.67	4.33	5.94	8.68	4.34	0.90	1.55	0.64
3			2.58	2.68	2.43	2.58	2.70	2.41	2.52	2.62	2.37	0.93	1.44	0.67
4			5.92	7.63	4.97	5.98	7.66	5.02	5.90	7.60	4.93	1.16	1.61	0.78
5			10.53	17.47	8.73	10.12	16.90	8.45	10.43	17.26	8.63	1.36	1.99	0.60
6			21.77	31.24	15.63	21.16	30.42	15.25	21.23	30.45	15.22	0.94	1.61	0.44
7	3	4+3=7	10.63	14.54	7.83	11.01	14.91	8.12	10.58	14.47	7.80	0.90	1.63	0.21
8			8.92	11.53	6.74	8.69	11.18	6.60	8.97	11.59	6.77	1.43	1.86	0.95
9			15.55	24.42	12.21	15.66	24.64	12.34	15.22	23.92	11.96	0.93	1.67	0.49
10			36.18	56.46	28.26	34.24	53.62	26.87	35.09	54.85	27.43	0.89	1.33	0.30
11			9.24	13.48	6.83	8.98	13.07	6.60	9.08	13.24	6.69	0.99	1.37	0.76
12			9.85	12.76	7.82	9.71	12.67	7.62	9.54	12.39	7.58	1.01	1.56	0.21
13			8.35	11.92	6.66	8.40	12.05	6.62	8.40	12.01	6.69	1.28	2.05	0.81
14			14.04	22.95	11.48	14.11	22.95	11.47	14.13	23.09	11.54	0.85	1.62	0.51
15			8.75	12.86	6.43	8.24	12.05	6.03	8.71	12.79	6.39	1.01	1.50	0.78
16			2.99	3.60	2.69	2.98	3.56	2.65	2.93	3.55	2.63	1.23	1.93	0.64
17			3.41	3.82	3.02	3.41	3.82	2.95	3.36	3.78	2.99	1.10	1.65	0.76
18	4	4+4=8	4.63	7.03	3.66	4.56	6.92	3.48	4.69	7.05	3.60	0.87	1.95	0.38
19			8.08	13.44	6.75	8.09	13.25	6.62	8.06	13.36	6.72	1.01	1.60	0.53
20			4.67	5.42	4.22	4.52	5.27	4.00	4.55	5.29	4.08	0.99	1.58	0.49
21			4.69	5.93	3.90	4.82	6.05	3.94	4.70	5.94	3.91	1.01	1.52	0.55
22	5	4+5=9	6.50	8.34	5.66	6.38	8.11	5.53	6.53	8.37	5.69	1.08	1.66	0.52
23			4.16	4.83	3.55	4.50	5.44	3.84	4.14	4.81	3.52	1.05	1.70	0.05
24			17.17	25.32	12.71	17.18	25.32	12.67	17.30	25.55	12.77	0.58	1.56	0.31
25			8.90	13.06	6.76	8.65	12.74	6.57	8.75	12.89	6.69	0.79	1.34	0.44
26			12.82	18.58	9.28	12.97	18.78	9.41	12.56	18.23	9.11	1.03	1.58	0.43
27			41.50	59.49	29.87	39.44	56.99	28.49	40.81	58.42	29.34	1.01	1.64	0.34
mean			10.82	15.65	8.38	10.60	15.36	8.20	10.67	15.43	8.26	1.00	1.62	0.51
SEM			1.79	2.73	1.31	1.70	2.61	1.25	1.75	2.67	1.28	0.03	0.03	0.04

Table S10: SUV values for the reconstructed PET_{CT}, PET_{MRI} and PET_{DL} images, and ADC values for the twenty-seven patients using ROI threshold of 50% for cancer in PZ and TZ.

Patients	Group	Gleason grade	Prostate cancer in PZ and TZ											
			Reconstructed PET images (SUV)									DWI ($\times10^{-3}$ mm^2 s^{-1})		
			CT_{med}	CT_{Kur}	CT_{Ske}	MR_{med}	MR_{Kur}	MR_{Ske}	DL_{med}	DL_{Kur}	DL_{Ske}	ADC_{med}	ADC_{Kur}	ADC_{Ske}
1	2	3+4=7	4.10	0.79	0.75	4.12	1.29	1.04	4.02	0.65	0.71	0.79	0.44	0.08
2			5.91	-0.66	0.62	5.62	-0.70	0.60	5.63	-0.67	0.62	0.89	0.23	0.79
3			2.59	-0.15	-0.73	2.58	-0.02	-0.61	2.53	-1.07	-0.79	0.87	1.62	1.06
4			5.85	0.38	0.71	5.92	0.49	0.74	5.83	0.85	0.75	1.16	-0.51	0.11
5			9.96	1.94	1.51	9.53	2.20	1.57	9.86	1.94	1.51	1.43	-0.79	-0.43
6			21.36	-1.01	0.29	20.77	-1.00	0.29	20.85	-1.01	0.90	0.91	0.87	0.70
7	3	4+3=7	10.49	-0.69	0.35	10.88	-0.71	0.33	10.40	-0.68	0.35	0.89	0.92	0.32
8			8.69	-0.99	0.37	8.46	-0.96	0.38	8.74	-0.99	0.37	1.44	-0.25	-0.40
9			14.83	0.04	0.90	14.96	0.06	0.91	14.51	0.03	0.90	0.89	0.81	0.82
10			34.87	0.46	1.02	33.06	0.48	1.01	33.84	0.47	1.02	0.87	-0.25	0.17
11			8.85	-0.52	0.67	8.59	-0.55	0.66	8.70	-0.52	0.67	0.97	1.51	1.08
12			9.52	-0.79	0.51	9.41	-0.84	0.47	9.21	-0.82	0.49	1.05	-0.18	-0.47
13			8.16	0.88	1.10	8.18	0.88	1.09	8.21	0.91	1.12	1.27	-0.08	0.41
14			13.20	2.02	1.63	13.31	2.12	1.64	13.26	2.02	1.63	0.84	1.10	0.99
15			8.61	-0.36	0.67	8.11	-0.39	0.65	8.55	-0.37	0.66	1.01	0.03	0.78
16			2.95	0.70	0.96	2.93	0.57	0.90	2.90	0.71	0.96	1.23	-0.63	0.26
17			3.38	-0.57	0.37	3.40	-0.25	-0.02	3.34	-0.54	0.39	1.10	0.09	0.66
18	4	4+4=8	4.51	0.71	0.88	4.43	0.10	0.77	4.59	0.56	0.81	0.87	1.98	1.36
19			7.83	4.72	1.97	1.89	4.60	1.85	7.83	4.73	1.96	0.99	-0.45	0.44
20			4.62	-0.07	0.76	4.47	0.03	0.75	4.49	-0.04	0.78	1.00	-0.99	-0.05
21			4.58	0.27	0.88	4.74	0.19	0.73	4.59	0.26	0.84	1.02	-1.04	0.14
22	5	4+5=9	6.38	0.04	0.84	6.25	0.00	0.78	6.41	0.05	0.84	1.08	-0.34	0.21
23			4.14	-0.39	0.34	4.47	-0.90	0.32	4.10	-0.34	0.35	1.04	0.11	-0.53
24			16.53	-0.66	0.59	16.53	-0.66	0.59	16.67	-0.66	0.59	0.58	1.65	1.35
25			8.80	0.98	0.87	8.56	0.98	0.89	8.65	1.02	0.90	0.78	0.06	0.63
26			12.46	-0.88	-0.42	12.64	-0.88	0.41	12.20	-0.87	0.43	1.03	0.28	0.43
27			40.23	-0.58	0.59	38.21	-0.56	0.61	39.62	-0.58	0.58	1.04	0.42	0.49
mean			10.49	0.20	0.70	10.07	0.20	0.71	10.35	0.25	0.75	1.00	0.24	0.42
SEM			1.73	0.23	0.10	1.67	0.23	0.09	1.69	0.23	0.09	0.03	0.15	0.10
